# Sex-specific associations between systolic, diastolic and pulse pressure and hemostatic parameters in the population-based KORA-Fit study: a cross-sectional study

**DOI:** 10.1186/s12959-023-00451-0

**Published:** 2023-01-19

**Authors:** J. Vogel von Falckenstein, D. Freuer, A. Peters, M. Heier, J. Linseisen, C. Meisinger

**Affiliations:** 1 Epidemiology, Faculty of Medicine, University of Augsburg, University Hospital of Augsburg, 86156 Augsburg, Germany; 2 Helmholtz Zentrum München, Institute for Epidemiology, Ingolstädter Landstr. 1, 85764 Neuherberg, Germany; 3 Institute for Medical Information Processing, Biometry and Epidemiology, Ludwig-Maximilians-Universität München, Munich, Germany; 4 German Center for Cardiovascular Disease (DZHK), Partner Site Munich Heart Alliance, Munich, Germany; 5 KORA Study Centre, University Hospital Augsburg, Augsburg, Germany

**Keywords:** Blood pressure, Hypertension, Coagulation, General population, KORA study

## Abstract

**Background:**

Several prior studies postulated an effect of hypertension on coagulation factors. However, population-based studies investigating the sex-specific associations between hypertension and hemostatic parameters are scarce. Therefore, we investigated the relationship between blood pressure and parameters of coagulation, namely activated partial thromboplastin time (aPTT), international normalized ratio (INR), fibrinogen, factor VIII, antithrombin III, protein C, protein S, and D-dimer in men and women from the general population.

**Methods:**

Based on 803 participants (376 men, 427 women) from the KORA-Fit Study the sex-specific relationship between systolic, diastolic, and pulse pressure and commonly measured coagulation factors were investigated using multivariable-adjusted linear regression models.

**Results:**

Hypertensive males had significantly higher median fibrinogen levels and factor VIII activity in comparison to normotensive males. There was a statistically significant difference between females with and without hypertension regarding the parameter fibrinogen, D-dimers, Protein S activity, and factor VIII activity. In multivariable linear regression analyses no significant association between systolic blood pressure, diastolic blood pressure, as well as pulse pressure and the investigated hemostatic parameters was found in men. In women, a significant positive association could be observed between systolic blood pressure and D-dimer level [β-estimate per mmHg increase 3.37 (95% CI 0.935–5.804; *p* = 0.007)] and between pulse pressure and D-dimer level [β-estimate per mmHg increase 5.351 (95% CI 1.772–8.930; *p* = 0.003)].

**Conclusions:**

It appears that sex differences exist in the association between blood pressure parameters and commonly measured coagulation markers in the general population. Further studies are needed to identify the underlying causes.

## Background

Worldwide, the prevalence of arterial hypertension is still high and it remains the greatest risk factor for mortality with 9.4 million deaths in 2010 [1] in industrialized countries. It is estimated that about one third of the 18 to 79 years old German population suffers from hypertension, and the frequency increases significantly with age [2]. Hypertension is recognized as a risk factor for the development of atherosclerosis via damage of the intima of blood vessels which leads to increased platelet aggregation [3]. Eventually, the coagulation system is activated, which may also stimulate the development of atherosclerosis. Thus, persons with hypertension are at increased risk of cardiovascular diseases, such as coronary heart disease (CHD), heart attack, heart failure, kidney failure, stroke and arterial occlusive disease [4, 5].

Previous studies, mostly from the 90 s, addressed the question whether hypertension confers a hypercoagulable state [6]. However, these investigations in subjects with and without hypertension led to conflicting results [7–10]. So far, epidemiological data from large population-based studies including men and women with and without hypertension and with the standardized measurement of blood pressure and a number of hematologic parameters are still scarce. Thus, the present study examines whether there are sex-specific associations between systolic and diastolic blood pressure and the parameters international normalized ratio (INR), activated partial thromboplastin time (aPTT), antithrombin III, fibrinogen, D-dimer, protein C, protein S, and factor VIII in a sample of the general adult population. Furthermore, in this context we also examined the role of pulse pressure, which is also a predictor of cardiovascular and mortality risk [11].

## Materials and methods

### Study sample

KORA (Cooperative Health Research in the Region of Augsburg, Germany) is a regional research platform for population-based studies. It consists of 4 cross-sectional baseline surveys (S1 1984/85, S2 1989/90, S3 1994/95 and S4 1999/2001) [12].

The KORA-Fit follow-up study was conducted from 22.01.2018 to 29.06.2019; all KORA participants born between 1945 and 1964 who agreed to be re-contacted were invited to the KORA study centre (*n* = 3059 or 64.4% of all appropriate participants).

In the present analysis, all S4 participants in KORA-Fit were considered (*n* = 1394 eligible persons). Of those, 856 participants took part in the KORA-Fit examination (61.4% of all eligible S4 persons), and citrate plasma samples were collected. For the present analysis, 803 participants (376 men, 427 women) with available data on hemostatic parameters were included.

The study methods were approved by the Ethics Committees of the Bavarian Chamber of Physicians (KORA-Fit EC No 17040). The study was performed in accordance with the Declaration of Helsinki. All study participants gave written informed consent.

### Data collection

During a face-to-face interview information on smoking habits, physical activity level, alcohol consumption, medication use, and socioeconomic status was gathered by trained and certified study nurses. Furthermore, the study participants underwent a standardized medical examination including collection of a fasting blood sample. All study participants should take their morning medications as usual prior to the study. Height and weight measurements were performed with the subjects in light clothing and without shoes; body mass index (BMI) was calculated as weight in kilograms divided by the height in m^2^. Education years were categorized into low (< 10 years of schooling) and high ( ≥10 years of schooling). Alcohol intake was classified into low risk (men ≤ 24 g/d, women ≤ 12 g/d) and risky consumption (men > 24 g/d, women > 12 g/d). The physical activity level was estimated by means of two separate four-category interview questions asking about the time per week spent on sports activities during leisure time in summer and winter. The winter and summer responses were combined to create one variable of leisure time physical activity [13]. A participant was defined as physically active during leisure time if he/she participated in sports in summer and winter and for more than one hour per week in at least one season [14]. In participants with or without hypertension, blood pressure was measured after a rest of at least 5 min at the right arm in the morning at the examination center. Three measurements were taken with 3 min intervals between the measurements and the results of the second and third measurements were averaged. Subjects who were aware of having hypertension, who were therefore taking antihypertensive medication and/or had blood pressure measurements of 140/90 mmHg or higher at the examination were defined as having actual hypertension. However, participants without known hypertension with normal blood pressure readings on examination but taking medications that affect blood pressure were not considered hypertensive.

Pulse pressure was calculated by building the difference between systolic and diastolic blood pressure. Anticoagulation therapy was defined as yes, if a participant was treated either with a novel oral anticoagulant (e.g. apixaban, rivaroxaban) or with phenprocoumon. Further information on the data collection procedures and examinations in the KORA studies have been described in detail elsewhere [15].

### Laboratory measurements

All hemostatic factors were measured in citrat plasma samples, which were collected in an overnight fasting state, and processed, aliquoted, and stored at -80° C until analysis. INR (reference value: 0.9–1.15) was calculated from the prothrombin ratio (Thromborel S, Siemens Healthcare). aPTT (reference value: 26–36 s) was measured photometrically (Pathromtin SL, Siemens Healthcare); antithrombin III activity (reference value: 78–113%) was determined by a chromogenic activity assay (Innovance Antithrombin-Assay, Siemens Healthcare). Fibrinogen (reference value: 210–400 mg/dl) was measured photometrically and turbidimetrically (Multifibren U, Siemens Healthcare). D-dimers (reference value: < 500 µg/L) were measured by a particle-enhanced immunoturbidimetric assay (Innovance D-dimer Kit, Siemens Healthcare). Protein-C and protein-S activities (reference values prot C: 70–140%, prot S men: 73–130%, women: 52–126%) were measured photometrically (Berichrom Protein C, Siemens Healthcare; Hemoclot Protein S). Factor VIII activity (reference value: 70–150%) was measured photometrically (Coagulation factor VIII Deficient Plasma reagents used with Pathromtin SL reagents, Siemens Healthcare). All measurements were performed on a Siemens BCS-XP analyzer (Siemens, Eschborn, Germany) except the measurement of protein S activity, which was measured on a CaoChrom analyzer (Wien, Austria).

Total cholesterol (reference value: ≤ 200 mg/dl) and HDL cholesterol (reference value: > 45 mg/dl) were measured enzymatically (Hoffmann-La Roche AG, Basel/Switzerland) on a Cobas 8000 c702 Roche chemistry analyzer. Non-HDL cholesterol (reference value: ≤ 130 mg/dl) was calculated by subtracting HDL cholesterol from total cholesterol.

### Statistical analysis

Continuous variables were checked for normal distribution by the Shapiro–Wilk test and were described by mean ± standard deviation (SD) in case of normal distribution; non-normally distributed variables were reported as median and interquartile range (IQR). Categorical variables were given as absolute frequencies and percentages. Medians of continuous variables were compared by the Mann–Whitney-U-Test, arithmetic means by the t-test, and the categorical variables by the Fisher’s exact test. The associations between the blood pressure exposures (systolic, diastolic, and pulse pressure) and the outcomes INR, aPTT, antithrombin III, fibrinogen, D-dimer, protein C, protein S, and factor VIII were investigated using multivariable linear regression models. The models were adjusted for age, waist circumference, leisure time physical activity, alcohol consumption, socioeconomic status, BMI, diabetes, smoking status, non-HDL cholesterol, and use of medications acting on blood pressure. Persons treated with anticoagulation drugs were excluded from the analyses with INR as outcome. We investigated whether the exposure-outcome associations were modified by sex or age by conducting formal tests for interaction (significance level 5%). Because there was a significant interaction with sex, separate analyses for men and women were carried out. All required model assumptions were ensured. Multicollinearity and autocorrelation were assessed by the variance inflation factor and Durbin-Watson statistics, respectively. The linearity assumption between continuous covariables and the respective outcome were tested using restricted cubic splines. The Breusch-Pagan test was applied to test for heteroscedasticity, and if present, robust standard errors were calculated. Influential observations were identified calculating Cook's distances (${D}_{i}$) and removed when ${D}_{i}>1$. P-values < 0.05 were considered as statistically significant. The statistical softwares IBM SPSS 26 and R (version 4.0.1) were used for data analysis.

## Results

Table 1 shows the sex-specific characteristics for participants with and without hypertension. Men and women with hypertension were significantly older than subjects without hypertension (mean age in men 64.1 vs 61.8 years; in women: 65.1 vs. 61.8 years; *p* < 0.0001 in both sexes). Mean systolic, diastolic, and pulse pressure values in men with hypertension were significantly higher than in normotensive men (systolic blood pressure: 136.0 vs. 122.8 mmHg; diastolic blood pressure: 78.9 vs. 74.0 mmHg; pulse pressure: 57.1 vs. 48.8 mmHg; *p* < 0.0001 for all comparisons). This was also the case in women (systolic blood pressure: 128.0 vs. 115.1 mmHg; diastolic blood pressure: 75.1 vs. 70.1 mmHg; pulse pressure: 52.9 vs. 45.1 mmHg; *p* < 0.0001 for all comparisons).

**Table 1:** Sex-specific characteristics given as means ± SD or n (%) for participants with and without hypertension

		**MEN**			**WOMEN**		
		HTN yes, *n* = 211	HTN no, *n* = 165	*p*-value	HTN yes, *n* = 170	HTN no, *n* = 257	*p*-value
Blood pressure systolic	mmHg	136.0 (16.4)	122.8 (9.7)	** < 0.0001**	128.0 (19.8)	115.1 (11.4)	** < 0.0001**
Blood pressure diastolic	mmHg	78.9 (10.3)	74.0 (7.3)	** < 0.0001**	75.1 (10.2)	70.1 (8.1)	** < 0.0001**
Pulse pressure	mmHg	57.1 (12.7)	48.8 (7.6)	** < 0.0001**	52.9 (13.6)	45.1 (7.9)	** < 0.0001**
Age	years	64.1 (5.6)	61.8 (5.8)	** < 0.0001**	65.1 (5.1)	61.8 (5.4)	** < 0.0001**
BMI	kg/m^2^	29.9 (5.6)	27.2 (3.5)	** < 0.0001**	29.6 (5.7)	26.1 (4.6)	** < 0.0001**
Education	education ≤ 10 yrs	70 (33.2)	47 (28.5)	0.370	89 (52.4)	82 (31.9)	** < 0.0001**
	education > 10 yrs	141 (66.8)	118 (71.5)		81 (47.6)	175 (68.1)	
Diabetes	yes	27 (12.8)	8 (4.8)	**0.011**	25 (14.8)	8 (3.1)	** < 0.0001**
	no	184 (87.2)	157 (95.2)		144 (85.2)	248 (96.9)	
Leisure time physical activity	yes	150 (71.1)	111 (67.3)	0.432	114 (67.1)	190 (73.9)	0.128
	no	61 (28.9)	54 (32.7)		56 (32.9)	67 (26.1)	
Smoking	Smoker	30 (14.2)	26 (15.8)	0.437	22 (12.9)	32 (12.5)	0.878
	Ex smoker	114 (54.0)	78 (47.3)		67 (39.4)	96 (37.4)	
	Never smoker	67 (31.8)	61 (37.0)		81 (47.6)	129 (50.2)	
Alcohol consumption	low risk	161 (76.3)	134 (81.7)	0.253	99 (58.2)	147 (57.2)	0.842
	risky	50 (23.7)	30 (18.3)		71 (41.8)	110 (42.8)	
Drugs acting on blood pressure	yes	160 (75.8)	8 (4.8)	** < 0.0001**	153 (90.0)	11 (4.3)	** < 0.0001**
	no	51 (24.2)	157 (95.2)		17 (10.0)	245 (95.3)	
Anticoagulative medication	yes	15 (7.1)	6 (3.6)	0.177	5 (2.9)	1 (0.4)	**0.039**
	no	196 (92.9)	159 (96.4)		165 (97.1)	255 (99.2)	
Total cholesterol	normal ≤ 200 mg/dl	113 (53.8)	77 (46.7)	0.212	67 (39.4)	65 (25.3)	**0.003**
	high > 200 mg/dl	98 (46.4)	88 (53.5)		103 (60.6)	192 (74.7)	
HDL cholesterol	low ≤ 45 mg/dl	69 (32.7)	41 (24.8)	0.110	19 (11.2)	7 (2.7)	**0.001**
	normal > 45 mg/dl	142 (67.3)	124 (75.2)		151 (88.8)	250 (97.3)	
non-HDL cholesterol	normal ≤ 130 mg/dl	89 (42.2)	56 (33.9)	0.110	55 (32.4)	82 (31.9)	1.000
	high > 130 mg/dl	122 (57.8)	109 (66.1)		115 (67.6)	175 (68.1)	

*HTN* Hypertension

Also, mean BMI was significantly higher in male and female participants with hypertension in comparison to normotensive subjects (men: 29.94 vs. 27.22; women: 29.59 vs. 26.12 mmHg; *p* < 0.0001 in both sexes). In both sexes, participants with hypertension suffered significantly more often from diabetes than subjects without hypertension. Females with hypertension more often were treated with anticoagulation drugs, and had higher total cholesterol as well as lower HDL cholesterol values in comparison to normotensive women. Other parameters did not differ significantly for men and women with and without hypertension (see Table 1).

The hemostatic parameters of men and women with and without hypertension are given in Table 2. In men, median fibrinogen levels and factor VIII activity were significantly higher in the hypertensive group than in normotensive participants (fibrinogen: 300.3 vs. 281.7 mg/dl, *p* = 0.006; factor VIII: 123.5 vs. 113.6%, *p* = 0.043). The other investigated hemostatic parameters did not significantly differ between hypertensive and non-hypertensive men (see Table 2).

**Table 2:** Coagulation factors of the subjects investigated (sex-specific values, that is median and IQR, for the total sample and stratified by hypertension yes/no)

(a) men							
**Coagulation factor**	**Overall (*n* = 378)**			**Hypertensive men**			***p*-Value**
			**Yes**		**No**		
Quick % (excl. Anticoag)	Median (IQR)	106.7 (100.1; 112.6)	Median (IQR)	106.8 (100.8; 113.5)	Median (IQR)	106.4 (99.4: 111.7)	0.284
INR in sec. (excl. Anticoag)	Median (IQR)	0.97 (0.93; 1.01)	Median (IQR)	0.97 (0.93; 1.00)	Median (IQR)	0.97 (0.94; 1.01)	0.307
aPTT (excl. Anticoag)	Median (IQR)	31.15 (29.3; 33.5)	Median (IQR)	31.0 (29.3; 33.2)	Median (IQR)	31.3 (29.3; 33.7)	0.586
Antithrombin III in mg/dl	Median (IQR)	99.2 (93.3; 106.2)	Median (IQR)	99.1 (92.5; 106.1)	Median (IQR)	99.2 (93.8; 106.3)	0.944
Fibrinogen in mg/dl	Median (IQR)	290.4 (258.3; 330.4)	Median (IQR)	300.3 (267.2; 342.6)	Median (IQR)	281.7 (252.9; 319.6)	**0.006**
D-dimers in µg/l	Median (IQR)	404.0 (303.8; 563.0)	Median (IQR)	420.0 (326.0; 576.0)	Median (IQR)	390.0 (288.5; 544.0)	0.162
Protein C in %	Median (IQR)	116.7 (107.3; 131.´3)	Median (IQR)	116.5 (107.1; 130.8)	Median (IQR)	116.8 (107.2; 131.8)	0.776
Protein S in %	Median (IQR)	131.9 (109.3; 157.2)	Median (IQR)	134.4 (113.3; 160.8)	Median (IQR)	129.1 (104.9; 153.5)	0.058
Factor VIII in %	Median (IQR)	119.1 (95.4; 141.7)	Median (IQR)	123.5 (97.0; 144.1)	Median (IQR)	113.6 (93.7; 138.6)	**0.043**
(b) women							
**Coagulation factor**	**Overall (*n* = 427)**			**Hypertensive women**			***p*-Value**
			**Yes**		**No**		
Quick % (excl. Anticoag)	Median (IQR)	110.4 (104.5; 115.8)	Median (IQR)	111.0 (104.4; 116.7)	Median (IQR)	110.3 (104.5; 115.1)	0.480
INR in sec. (excl. Anticoag)	Median (IQR)	0.94 (0.91; 0.98)	Median (IQR)	0.94 (0.91; 0.98)	Median (IQR)	0.95 (0.92; 0.98)	0.558
aPTT (excl. Anticoag)	Median (IQR)	30.2 (28.4; 32.6)	Median (IQR)	30.1 (28.3; 32.7)	Median (IQR)	30.3 (28.5; 32.4)	0.998
Antithrombin III in mg/dl	Median (IQR)	104.7 (98.3; 110.8)	Median (IQR)	103.8 (97.1; 109.6)	Median (IQR)	105.6 (99.2; 112.8)	0.062
Fibrinogen in mg/dl	Median (IQR)	300.3 (263.5; 341.0)	Median (IQR)	317.1 (266.8; 356.7)	Median (IQR)	292.4 (259.8; 325.5)	**0.001**
D-dimers in µg/l	Median (IQR)	406.0 (306.0; 554.0)	Median (IQR)	459.5 (353.0; 684.8)	Median (IQR)	370.0 (282.0; 505.0)	** < 0.0001**
Protein C in %	Median (IQR)	129.0 (116.2; 142.1)	Median (IQR)	128.7 (116.6; 147.1)	Median (IQR)	129.4 (115.2; 140.2)	0.405
Protein S in %	Median (IQR)	120.1 (101.3; 138.9)	Median (IQR)	124.8 (106.5; 145.1)	Median (IQR)	117.9 (99.4; 133.2)	**0.008**
Factor VIII in %	Median (IQR)	123.2 (101.1; 143.4)	Median (IQR)	125.7 (105.4; 152.1)	Median (IQR)	118.7 (97.8; 140.8)	**0.031**

In women, there was a statistically significant difference between participants with and without hypertension regarding the parameters fibrinogen, D-dimers, protein S activity and factor VIII activity; females with hypertension showed higher levels of these parameters (fibrinogen: 317.1 vs. 292.4 mg/dl, *p* = 0.001; D-dimers: 459.5 vs. 370.0 µg/l, *p* = < 0.001; protein S: 124.8 vs. 117.9%, *p* = 0.008; factor VIII: 125.7 vs. 118.7%, *p* = 0.031). Other hemostatic parameters did not differ significantly in women (see Table 2).

### Linear regression analyses

In multivariable-adjusted linear regression analyses (see Table 3) a significant positive association could be observed between systolic blood pressure and D-dimer level [β-estimate per mmHg increase 3.37 (95% CI 0.935–5.804; *p* = 0.007)] and between pulse pressure and D-dimer level [β-estimate per mmHg increase 5.351 (95% CI 1.772–8.930; *p* = 0.003)] in women. No further significant associations were observed in women. Furthermore, no notable associations between the three exposures and the investigated coagulation factors were found in men.

**Table 3:** Association between blood pressure measurements and coagulation factors. Results of the multivariable linear regressions in men and women

**MEN**	**Systolic BP**		**Diastolic BP**		**Pulse pressure**	
	ß-estimate (95% CI)	*P*-value	ß-estimate (95% CI)	*P*-value	ß-estimate (95% CI)	*P*-value
INR	0 (-0.001, 0)	0.497	0 (-0.001, 0.001)	0.942	0 (-0.001, 0)	0.394
aPTT (sec)	0.012 (-0.01, 0.034)	0.289	0 (-0.037, 0.038)	0.987	0.021 (-0.009, 0.051)	0.161
Protein C activity (%)	0.043 (-0.077, 0.163)	0.483	-0.041 (-0.243, 0.161)	0.69	0.108 (-0.054, 0.271)	0.192
Protein S activity (%)	-0.02 (-0.254, 0.214)	0.868	-0.047 (-0.438, 0.343)	0.812	-0.004 (-0.321, 0.313)	0.980
Antithrombin III (mg/dl)	0.012 (-0.058, 0.081)	0.739	0.01 (-0.107, 0.126)	0.868	0.014 (-0.08, 0.108)	0.768
Fibrinogen (mg/dl)	-0.185 (-0.592, 0.223)	0.374	-0.145 (-0.837, 0.548)	0.682	-0.255 (-0.805, 0.294)	0.362
D-dimers µg/l	-1.941 (-4.674, 0.793)	0.164	-0.501 (-5.108, 4.107)	0.831	-3.030 (-6.713, 0.653)	0.107
Factor VIII activity (%)	0.045 (-0.186, 0.277)	0.702	0.182 (-0.206, 0.569)	0.358	-0.038 (-0.349, 0.275)	0.816
**WOMEN**	**Systolic BP**		**Diastolic BP**		**Pulse pressure**	
	ß-estimate (95% CI)	*P*-value	ß-estimate (95% CI)	*P*-value	ß-estimate (95% CI)	*P*-value
INR	0.000 (0.000, 0.000)	0.896	0.000 (0.000, 0.001)	0.461	0.000 (-0.001, 0.000)	0.651
aPTT (sec)	-0.003 (-0.022, 0.016)	0.759	0.02 (-0.014, 0.054)	0.253	-0.02 (-0.049, 0.008)	0.164
Protein C activity (%)	0.065 (-0.042, 0.173)	0.233	0.127 (-0.063, 0.317)	0.19	0.055 (-0.103, 0.213)	0.492
Protein S activity (%)	0.024 (-0.154, 0.202)	0.795	-0.033 (-0.332, 0.267)	0.831	0.072 (-0.180, 0.324)	0.576
Antithrombin III (mg/dl)	-0.02 (-0.077, 0.038)	0.506	-0.044 (-0.143, 0.055)	0.385	-0.012 (-0.098, 0.075)	0.793
Fibrinogen (mg/dl)	-0.24 (-0.612, 0.132)	0.206	-0.341 (-1.004, 0.322)	0.313	-0.291 (-0.836, 0.255)	0.296
D-dimers (µg/l)	**3.37 (0.935, 5.804)**	**0.007**	2.718 (-1.614, 7.051)	0.218	**5.351 (1.772, 8.930)**	**0.003**
Factor VIII activity (%)	-0.029 (-0.235, 0.177)	0.785	-0.081 (-0.446, 0.283)	0.662	-0.004 (-0.307, 0.299)	0.978

Exposures: systolic, diastolic, and pulse pressure; Outcomes: INR, aPTT, protein C, protein S, antithrombin III, fibrinogen, D-dimers, Factor VIII. Level of significance at *P* < 0.05. *ß* regression coefficient, *CI* Confidence interval, *BP* Blood pressure

Adjusted for age, BMI, diabetes, smoking status, non HDL-Cholesterol, and medications acting on blood pressure. Participants with anticoagulative medication were excluded from the linear regression models

## Discussion

The present observational study explored the sex-specific association between hypertension and a number of commonly measured coagulation factors. It was found that fibrinogen levels and factor VIII activity differed significantly between normotensive and hypertensive men. Hypertensive women had statistically significantly higher levels of fibrinogen, D-dimers, protein S activity and factor VIII activity in comparison to normotensive women. In multivariable linear regression analysis only the associations between systolic blood pressure and pulse pressure and D-dimer in women remained statistically significant, while no other significant results were found in both sexes.

Available literature reported that hypertension is associated with a hypercoagulable state [6], which may contribute to the pathogenesis of atherothrombotic diseases [16]. Decades ago, Letcher et al. found that in haematocrit-matched hypertensives, the levels of fibrinogen are increased, a change that might partly be responsible for a higher blood viscosity [17]. However, a number of early epidemiological studies showed only weak associations [10, 18, 19]. Most prior studies could show that individuals with hypertension had higher fibrinogen levels than normotensive persons [7, 20], and that women have higher fibrinogen levels than men [7, 21, 22], a finding which could be confirmed by our study. Some former investigations reported no independent association between blood pressure and fibrinogen levels after multivariable adjustment for other cardiovascular risk factors [10, 23, 24]. Other studies [9, 18] showed a weak but independent association in women only. For example, the association between fibrinogen levels and blood pressure was investigated in the population-based Northern Sweden MONICA study including 1558 men and women aged 25 to 64 years [9]. However, it remained unclear whether there are sex differences in relationships between blood pressure and fibrinogen levels [9, 10, 18, 23]. In the present population-based study no independent association between blood pressure and fibrinogen levels in multivariable-adjusted regression analysis was observed, neither in men nor in women.

Factor VIII is mainly synthesized in hepatocytes, but also endothelial cells, kidneys, and lymphatic tissue [25]. In the blood-stream it is present in a non-covalent complex in association with the von Willebrand factor [26, 27]. We found significantly higher factor VIII levels in men and women with hypertension in comparison to normotensive individuals. In the population-based third MONICA Glasgow survey, factor VIII was correlated with diastolic blood pressure in men but not women in age-adjusted Spearman rank correlation analysis [28]. However, in our study in both sexes the results of the multivariable linear regression models do not support an independent association between systolic and diastolic blood pressure as well as pulse pressure and factor VIII levels. To the best of our knowledge, no population-based studies have investigated the association between blood pressure and factor VIII in detail. There are only a few studies on the association between von Willebrand factor and blood pressure, and they found no independent relationship [29]. Unfortunately, no von Willebrand factor measurements were available in our study. Further investigations on the association between blood pressure and factor VIII in the general population are necessary.

Protein S and protein C are vitamin K dependent inhibitors of blood coagulation [30]. Contrary to protein C, which is synthesized in the liver only, protein S is synthesized in a number of cells including endothelial cells. In this study, significantly higher protein S levels were found in women with than without hypertension. Prior studies investigating the relationship between protein S and blood pressure are scarce. In the third Glasgow MONICA survey an age-adjusted Spearman rank correlation between protein S levels and blood pressure was found in both, men and women [28]. However, in regression analysis, in both sexes there was no significant association [28]. In another study blood protein S levels were higher in relatives of hypertensive men than in men without a family history of hypertension [31].

Plasma D-dimer is a degradation product of cross-linked fibrin and a marker of hypercoagulability and thrombosis [32]. Higher fibrin D-dimer concentrations may reflect an increased turnover of fibrin [18]. Moderately high levels of D-dimer have been associated with an increased risk of subsequent thrombotic events, particularly in patients with prior vascular disease [32]. Serum D-dimer levels correlate with the extent of the total thrombolytic activity [32]. In our study, the D-dimer levels were significantly higher in hypertensive women compared to non-hypertensive ones, but this difference was not seen in men. Higher D-dimer levels in women compared to men were also reported from an Italian study [33]. Linear regression models attempting to assess the independent association between the systolic, diastolic and pulse pressure showed a significant relationship between systolic blood pressure and pulse pressure and D-dimer levels in women only. Our finding is in accordance with results from the Edinburgh Artery Study [34], in which systolic blood pressure in women was independently associated with D-dimer levels. Higher fibrin D-dimer levels in hypertensive than normotensive patients have been shown in further studies [33, 35].

The strengths of the present study are primarily the population-based design, and the availability of laboratory data, information on medication intake, and standardized assessed cardiovascular risk factors, including standardized blood pressure measurement. The sample size allowed for a sufficiently powered sex-specific analysis. This study also has limitations. Because the analyses were based on a follow-up examination of the population-based KORA study, it could be argued that the responders are not representative of the initial population-based sample. Thus, selection bias that may have affected the present results cannot be excluded. Furthermore, residual confounding by unmeasured variables cannot be entirely ruled out. The cross-sectional design of the study, the evaluation of leisure physical activity by self-report only, and the missing information on other types of physical activity represent further shortcomings [36]. Finally, because this study included German subjects born between 1945 and 1964, the results are not transferable to other age-groups and persons of other ethnicity.

In conclusion, while in both sexes there were significant differences in fibrinogen and factor VIII levels between hypertensive versus non-hypertensive subjects, the blood levels of protein S and D-dimers in hypertensives versus non-hypertensives differed only in women. There was no significant association between systolic, diastolic, and pulse pressure and any of the coagulation factors in both sexes, except for an independent association between systolic and pulse pressure and D-dimers in women. Thus, it seems that sex differences exist in the association between blood pressure parameters and commonly measured coagulation markers in the general population. Further studies are needed to identify the underlying causes.

## Data Availability

The data that support the findings of this study are available from Helmholtz Zentrum München but restrictions apply to the availability of these data, which are not publicly available. Data are however available from the authors upon reasonable request and with permission of Helmholtz Zentrum München.

## Abbreviations

aPTT: Activated partial thromboplastin time
BMI: Body mass index
CHD: Coronary heart disease
HDL: High-densitiy lipoprotein
INR: International normalized ratio
IQR: Interquartile range
KORA: Cooperative Health Research in the Region of Augsburg, Germany
SD: Standard deviation
